# Determinants of excessive daytime sleepiness in two First Nation communities

**DOI:** 10.1186/s12890-017-0536-x

**Published:** 2017-12-12

**Authors:** Ina van der Spuy, Chandima P. Karunanayake, James A. Dosman, Kathleen McMullin, Gaungming Zhao, Sylvia Abonyi, Donna C. Rennie, Joshua Lawson, Shelley Kirychuk, Judith MacDonald, Laurie Jimmy, Niels Koehncke, Vivian R. Ramsden, Mark Fenton, Gregory P. Marchildon, Malcolm King, Punam Pahwa

**Affiliations:** 1 School of Physical Therapy, University of Saskatchewan, 104 Clinic Place, Saskatoon, SK S7N 2Z4 Canada; 2 Canadian Centre for Health and Safety in Agriculture, University of Saskatchewan, 104 Clinic Place, Saskatoon, SK S7N 2Z4 Canada; 3 Department of Medicine, College of Medicine, University of Saskatchewan, 103 Hospital Drive, Saskatoon, SK S7N 0W8 Canada; 4 First Nations University of Canada, Prince Albert Campus, 1301 Central Avenue, Prince Albert, SK S6V 4W1 Canada; 5 Department of Community Health and Epidemiology, College of Medicine, University of Saskatchewan, 107 Wiggins Road, Saskatoon, SK S7N 5E5 Canada; 6 College of Nursing, University of Saskatchewan, 104 Clinic Place, Saskatoon, SK S7N 2Z4 Canada; 7 Community A, Duck Lake, Saskatoon, SK Canada; 8 Community B, Montreal Lake, Saskatoon, SK Canada; 9 Department of Academic Family Medicine, University of Saskatchewan, West Winds Primary Health Centre, 3311 Fairlight Drive, Saskatoon, SK S7M 3Y5 Canada; 10 Division of Respirology, Critical Care and Sleep Medicine, Department of Medicine, College of Medicine, University of Saskatchewan, 103 Hospital Drive, Saskatoon, SK S7N 0W8 Canada; 11 Institute of Health Policy, Management and Evaluation, University of Toronto, Suite 425, 155 College Street, Toronto, ON M5T 3M6 Canada; 12 Faculty of Health Sciences, Simon Fraser University, 8888 University Drive, Burnaby, BC V5A 1S6 Canada

**Keywords:** First Nations, Indigenous, Sleepiness, Epworth sleepiness scale, Co-morbidities, Poverty, Snoring

## Abstract

**Background:**

Excessive daytime sleepiness may be determined by a number of factors including personal characteristics, co-morbidities and socio-economic conditions. In this study we identified factors associated with excessive daytime sleepiness in 2 First Nation communities in rural Saskatchewan.

**Methods:**

Data for this study were from a 2012–13 baseline assessment of the First Nations Lung Health Project, in collaboration between two Cree First Nation reserve communities in Saskatchewan and researchers at the University of Saskatchewan. Community research assistants conducted the assessments in two stages. In the first stage, brochures describing the purpose and nature of the project were distributed on a house by house basis. In the second stage, all individuals age 17 years and older not attending school in the participating communities were invited to the local health care center to participate in interviewer-administered questionnaires and clinical assessments. Excessive daytime sleepiness was defined as Epworth Sleepiness Scale score > 10.

**Results:**

Of 874 persons studied, 829 had valid Epworth Sleepiness Scale scores. Of these, 91(11.0%) had excessive daytime sleepiness; 12.4% in women and 9.6% in men. Multivariate logistic regression analysis indicated that respiratory comorbidities, environmental exposures and loud snoring were significantly associated with excessive daytime sleepiness.

**Conclusions:**

Excessive daytime sleepiness in First Nations peoples living on reserves in rural Saskatchewan is associated with factors related to respiratory co-morbidities, conditions of poverty, and loud snoring.

## Background

Excessive daytime sleepiness (EDS) is a common problem in various populations [1–5]. It has been associated with age [3, 5], sex [5–7], body mass index (BMI) [8–10], shift work [11], snoring [12–14], comorbidities [15], social conditions [5, 16] and obstructive sleep apnea [2, 17, 18]. Deleterious outcomes of EDS include poor work performance [19], motor vehicles accidents [20, 21], and work injuries [22, 23]. We have recently observed EDS prevalence of 15.9% to 20.8% in Caucasian populations in rural Saskatchewan [5, 24]. High rates of EDS have been documented in New Zealand Maori Indigenous populations (21.3%) compared to New Zealand non-Maori populations (13.9%) [25]. Knowledge is limited for First Nations populations.

First Nations people in Canada characteristically have high rates of respiratory morbidities, including asthma and chronic obstructive pulmonary disease (COPD) [26–30]. Among First Nations people living on reserves in Canada, 59% engaged in non-traditional use of tobacco (smoking) [31]. Age standardized hospital separation rates in Western Canada for Indigenous people for all respiratory diseases in 2000 were high at 3040/100,000 compared to 920/100,000 in the general population [27].

First Nations populations in Canada are characterized by disadvantaged socio-economic status [32]. The average annual income in 2010 was $18,586 for on-reserve First Nations people compared to $41,052 for other Canadians [32]. Twenty-eight percent of on-reserve First Nations persons live in crowded housing conditions of more than one person per room, compared to 4% of non-Indigenous persons in Canada [33]. Forty-three percent of persons living on-reserves had homes in need of major repairs [33]. Poor housing conditions are associated with dampness and mold which may be associated with deleterious respiratory outcomes [27].

In Canada, the term commonly used to refer to people registered under the Indian Act is “First Nations” [34]. There is limited information on EDS among First Nations people in Canada [35]. In 2011, of the 1,033,381 persons in Saskatchewan, 103,205 were First Nations [36]. The objective of this study was to identify the prevalence of EDS and to investigate factors contributing to EDS among adult First Nations people living in two reserve communities in rural Saskatchewan.

## Methods

### Study sample

The data for this study are from a 2012–13 baseline assessment of the First Nations Lung Health Project which was conducted as part of a research collaboration between two Cree First Nation communities in Saskatchewan and researchers at the University of Saskatchewan [37]. The sampling frame was all currently residing community members based on 321 households with 810 adults in Community A, and 259 households with 760 adults in Community B [37]. Baseline data from 432 adults living in 173 households (53.9% household participation rate) in Community A and 442 adults living in 233 households (89.9% household participation rate) in Community B were obtained. Of 874 persons studied, 829 had valid Epworth Sleepiness Scale scores obtained from participants aged 17 years and older.

### Data acquisition

The study coordinator (KM) is from the LaRonge Cree First Nation. The clinical aspects of the study were conducted primarily by students from the communities who were attending post-secondary institutions and who were available during the summer holiday period. The students were attending nursing, education and social work training courses during the school year, and were trained in the techniques involved at the base laboratory of the Canadian Centre for Health in Agriculture prior to the commencement of the study. They were supervised by the project coordinator and other research staff. The baseline assessment was conducted in two stages. The rationale and purpose of the study were determined with representatives of the communities including Elders, community leadership, health provider personnel and students. The first stage consisted of introducing the project to the community by meetings and brochures. In the second stage, all individuals age 17 years and older not attending school were invited to the community health care center for interviewer-administered questionnaires and participation in clinical assessments. The degree of sleepiness in individuals was based on their response to Epworth Sleepiness Scale (ESS) questionnaire [38–41]. ESS shows good reliability and validity [39, 41]. We used an ESS score of 11 to 24 as indicating high ESS score [40] which we considered the principal outcome. Independent variables of interest were self-reported age, sex, height, weight, (BMI, kg/m^2^) (overweight BMI = 25–29.9; obese BMI ≥ 30), education level (completed post-secondary, completed university, completed high school, less than high school) marital status, non-traditional use of tobacco (smoking) (current, former, never) and alcohol use. “Doctor ever diagnosed” conditions included sinus trouble, heart problem, tuberculosis, attack of bronchitis, emphysema, chronic bronchitis, COPD and asthma. Other factors elicited by the questionnaire included chronic cough/chronic phlegm and shortness of breath (SOB), loud snoring, money at end of month, annual household income level, state of house repairs, and number of persons per room as an index of crowding. For the analysis, we used the term “chronic lung disease” to include one or more of emphysema, chronic bronchitis, chronic cough/chronic phlegm and COPD.

### Statistical analysis

Statistical analysis was conducted using SAS version 9.4© (SAS Institute Inc., Cary, NC, USA).

Logistic regression models were used to assess relationships between ESS and covariates. A multilevel logistic regression model by generalized estimating equations approach was used to fit the model with individuals (first level) clustering within households (second level). The significant contribution of potential risk factors, confounders and interactive effects was determined by fitting a series of multilevel models. Variables with *p* < 0.20 in univariate analysis became candidates for the multivariable model. The variables retained in the final multivariable model included those that were statistically significant (i.e. *p* < 0.05) as well as sex, non-traditional use of tobacco, and BMI. Odds ratios (ORs) and 95% confidence intervals (CIs) were used to present the strength of associations.

## Results

The mean (±SD) age of the 408 men who participated was 33.7 (±13.9) years and of the 421 women was 35.9 (±14.4) years. Fifty percent had completed high school (Grade 12). Twenty-nine percent were overweight and 35% were obese. Non-traditional use of tobacco (current smoking) was present in 78.3%, ex-smoking in 12.2%, and never smoking in 9.5% persons. Loud snoring was reported by 17.0% men and 14.8% women. There were 91 (11.0%) adults with an ESS score > 10. More women (12.4%) than men (9.6%) had ESS scores >10.

As shown in Table 1, unadjusted univariate analysis showed that older age [>55 years of age, OR 4.46 (95% CI 2.34 to 8.52)] was significantly associated with high ESS score. With regard to doctor diagnosed conditions significantly associated with the risk of having high ESS scores included: sinus trouble [OR 2.24 (95% CI 1.42 to 3.54)], heart problems [OR 2.55 (95% CI 1.47 to 4.41)], tuberculosis [OR 2.71 (95% CI 1.30 to 5.63)], attack of bronchitis [OR 2.22 (95% CI 1.38 to 3.56)], chronic lung disease [OR 2.35 (95% CI 1.51 to 3.64)], and SOB [OR 3.15 (95% CI 1.92 to 5.18)]. Loud snoring [OR 1.68 (95% CI 1.02 to 2.77)] was also associated with high ESS scores. House in need of repairs and crowding (more than one person per room) were significant at *p* < 0.2 and hence included in the multivariate model as potential confounders.

**Table 1:** Univariate associations between ESS score > 10 and independent variables of interest^a^

Variables	ESS Score, *n* (%)		Univariate OR (95% CI)
	Normal (*n* = 738)	Abnormal (*n* = 91)	
Demographics			
Sex			
Female (reference)	369 (87.6)	52 (12.4)	1.00
Male	369 (90.4)	39 (9.6)	0.76 (0.48–1.20)
Age, in years			
17–25 (reference)	266 (92.7)	21 (7.3)	1.00
26–35	180 (88.2)	24 (11.8)	1.71 (0.90–3.26)
36–45	119 (89.5)	14 (10.5)	1.45 (0.69–3.03)
46–55	113 (91.1)	11 (8.9)	1.30 (0.58–2.90)
> 55	**60 (74.1)**	**21 (25.9)**	**4.46 (2.34–8.52)**
Educational level			
Completed postsecondary education (reference)	103 (88.8)	13 (11.2)	1.00
Completed university	89 (88.1)	12 (11.9)	1.02 (0.41–2.53)
Completed high school	191 (92.7)	15 (7.3)	0.61 (0.26–1.43)
Less than high school	354 (87.6)	50 (12.4)	1.12 (0.56–2.25)
Marital status			
Widowed/divorced/separated/single (reference)	430 (88.5)	56 (11.5)	1.00
Married/common in law	288 (89.4)	34 (10.6)	0.88 (0.56–1.38)
Body mass index, kg/m^2^			
Normal (<25) (reference)	251 (90.0)	28 (10.0)	1.00
Overweight (25–29.9)	213 (88.4)	28 (11.6)	1.14 (0.64–2.05)
Obese (≥30)	258 (88.4)	34 (11.6)	1.15 (0.68–1.95)
Smoke status			
Non-smoker (reference)	68 (86.1)	11 (13.9)	1.00
Ex-smoker	85 (84.2)	16 (15.8)	1.27 (0.51–3.16)
Current smoker	585 (90.1)	64 (9.9)	0.72 (0.36–1.42)
In past 12 months, had ≥5 drinks on one occasion			
No (reference)	156 (88.1)	21 (11.9)	1.00
Yes	579 (89.6)	67 (10.4)	0.84 (0.50–1.41)
Employment status			
Yes (reference)	388 (89.2)	47 (10.8)	1.00
No	346 (88.7)	44 (11.3)	1.05 (0.67–1.64)
Doctor ever diagnosed:			
Sinus trouble			
No (reference)	470 (91.8)	42 (8.2)	1.00
Yes	**189 (83.3)**	**38 (16.7)**	**2.24 (1.42–3.54)**
Heart problem			
No (reference)	674 (90.2)	73 (9.8)	1.00
Yes	**62 (78.5)**	**17 (21.5)**	**2.55 (1.47–4.41)**
Tuberculosis			
No (reference)	597 (90.5)	63 (9.5)	1.00
Yes	**43 (78.2)**	**12 (21.8)**	**2.71 (1.30–5.63)**
Attack of bronchitis			
No (reference)	466 (91.6)	43 (8.4)	1.00
Yes	**180 (82.9)**	**37 (17.1)**	**2.22 (1.38–3.56)**
Ever had asthma			
No (reference)	616 (89.3)	74 (10.7)	1.00
Yes	122 (87.8)	17 (12.2)	1.12 (0.64–1.95)
Questionnaire ascertained conditions			
Chronic Lung Disease^b^			
No (reference)	554 (91.6)	51 (8.4)	1.00
Yes	**184 (82.1)**	**40 (17.9)**	**2.35 (1.51–3.64)**
SOB			
No (reference)	367 (94.3)	22 (5.7)	1.00
Yes	**365 (84.1)**	**69 (15.9)**	**3.15 (1.92–5.18)**
Loud snoring			
No (reference)	626 (89.9)	70 (10.1)	1.00
Yes	**110 (84.0)**	**21 (16.0)**	**1.68 (1.02–2.77)**
Socio-economic Status			
Money left over at end of the month			
Some (reference)	188 (86.2)	30 (13.8)	1.00
Just enough	171 (90.5)	18 (9.5)	0.64 (0.34–1.22)
Not enough	340 (90.2)	37 (9.8)	0.65 (0.37–1.15)
Annual household income			
$20,000 and over (reference)	199 (91.7)	18 (8.3)	1.00
$10,000–$19,999	106 (86.9)	16 (13.1)	1.58 (0.76–3.27)
Less than $10,000	188 (85.8)	31 (14.2)	1.75 (0.88–3.47)
Refusal/not stated	245 (90.4)	26 (9.6)	1.09 (0.54–2.19)
Home need repairing			
No (reference)	205 (89.5)	24 (10.5)	1.00
Minor	188 (85.1)	33 (14.9)	1.51 (0.82–2.80)
Major	188 (85.1)	28 (8.7)	0.81 (0.46–1.43)
Home crowded status (person per room)			
one or less person per room (reference)	188 (85.1)	52 (9.7)	1.00
More than one person per room	215 (86.3)	34 (13.7)	1.44 (0.88-2.36)

^a^Row percentages are presented

^b^“Chronic Lung Disease” includes groups of chronic cough, chronic phlegm, chronic bronchitis, emphysema and chronic obstructive pulmonary disease

Multivariable logistic regression analysis (Table 2) indicated a trend towards statistical significance for age greater than 55 years [OR 2.27 (95% CI 0.94 to 5.52); *p* = 0.07]. Doctor ever diagnosed tuberculosis [OR 3.02 (95% CI 1.32 to 6.87)] was significantly related to high ESS. Chronic lung disease [OR 2.01 (95% CI 1.15 to 3.50)] and SOB [OR 2.76 (95% CI 1.52 to 4.99)] were significantly related to high ESS scores. Loud snoring [OR 2.21 (95% CI 1.13 to 4.33)] continued to be an associated condition. Socio-economic factors of low income [OR 2.59 (95% CI 1.20 to 5.60)], house in need of repairs [OR 2.40 (95% CI 1.18 to 4.87)] and more than one person per room [OR 2.07 (95% CI 1.16 to 3.69)] were significantly associated with high ESS scores. There were no associations for BMI and smoking with high ESS scores.

**Table 2:** Multivariable logistic regression of the association between ESS score > 10 and independent variables of interest

Variables	Multivariate OR (95% CI)	*P*-value
Sex		
Female (reference)	1.00	
Male	0.67 (0.36–1.22)	0.189
Age, in years		
17–25 (reference)	1.00	
26–35	2.06 (0.98–4.35)	0.057
36–45	1.17 (0.49–2.82)	0.722
46–55	0.84 (0.34–2.07)	0.709
> 55	2.27 (0.94–5.52)	0.070
Body mass index, kg/m2		
Normal (<25) (reference)	1.00	
Overweight (25–29.9)	0.75 (0.38–1.49)	0.409
Obese (> = 30)	0.49 (0.23–1.03)	0.061
Smoke status		
Non-smoker (reference)	1.00	
Ex-smoker	1.37 (0.44–4.24)	0.586
Current smoker	0.52 (0.21–1.29)	0.159
Tuberculosis		
No (reference)	1.00	
Yes	**3.02 (1.32–6.87)**	**0.009**
Chronic Lung Disease		
No (reference)	1.00	
Yes	**2.01 (1.15–3.50)**	**0.014**
Shortness of breath		
No (reference)	1.00	
Yes	**2.76 (1.52–4.99)**	**0.001**
Loud snoring		
No (reference)	1.00	
Yes	**2.21 (1.13–4.33)**	**0.021**
Annual household income		
More than $20,000 (reference)	1.00	
$10,000–$19,999	1.93 (0.84–4.41)	0.119
Less than $10,000	**2.59 (1.20–5.60)**	**0.016**
Refusal/not stated	1.37 (0.61–3.08)	0.450
Home need repairing		
No (reference)	1.00	
Minor	**2.40 (1.18–4.87)**	**0.015**
Major	0.90 (0.47–1.75)	0.766
Home crowded status (person per room)		
one or less person per room (reference)	1.00	
More than one person per room	**2.07 (1.16–3.69)**	**0.014**

## Discussion

The findings from this study highlight the strong associations of certain respiratory co-morbidities, indicators of poverty and snoring with EDS among people living on two on-reserve First Nation communities in the Province of Saskatchewan in Canada.

In addition, annual household income (less than $10,000 per year), houses in need of repairs, and over-crowding, all indicators of social determinants of health consistent with poverty [42–44] were associated with high ESS scores. The relationship of poverty to sleep disturbance has been described for non-Indigenous populations [45–47]. Social determinants of health of which low socio-economic status is one, are associated with co-morbidities [48–50]. Respiratory co-morbidities associated with EDS in the present study were doctor ever diagnosed tuberculosis, reported chronic lung disease, and SOB. The significant relationship between SOB and EDS in our study is consistent with the observations of Kaneita et al. [51] The association between EDS and chronic lung disease in our study is consistent with the findings of Karachaliou et al. [52] and Koutsourelakis et al. [53] We did not find reports linking past tuberculosis with high ESS score. However, chronic lung disease has been observed in former tuberculosis patients [54], and it is possible that the apparent relationship between past tuberculosis and high ESS score in our study is as a result of chronic lung disease in former tuberculosis patients.

In the univariate analysis, older age (≥55 years of age) was associated with high ESS score. In the multivariate analysis borderline significance for age persisted. Increasing age is recognized as being associated with increased risk for EDS [3, 5]. The prevalence of high ESS scores in this fairly young population (men 33.7 years, women 35.9 years) that we studied was 11.0%. The prevalence of high ESS score (25.9%) in the oldest (>55 year) age group was higher than in the non-Indigenous population (18.5%) of rural Saskatchewan described by Gjevre et al. [5], and more like that described by Paine et al. for the Maori population (21.3%) in New Zealand [25].

Obesity has been identified as an independent predictor of EDS in both men and women.^9,^ [55, 56] However, in our study the effect of overweight and obesity on high ESS scores was not statistically significant, likely due to lack of variability with 64% of the population being either overweight or obese. Following discussion with the communities, the high percentage of obesity can be addressed in future by the introduction of healthier eating and exercise programs.

The significance of EDS for the health of the populations participated is important to consider. EDS is a hallmark feature of obstructive sleep apnea [2, 17, 18]. Among those with high ESS scores in the populations that we studied, there may be a high prevalence of undiagnosed and untreated sleep disordered breathing with all of the implications for cardiovascular outcomes [57, 58] and accidents [22, 59, 60].

Mechanisms by which these factors might affect EDS merit discussion. We have shown that socio-economic factors relate to respiratory status in rural people [61]. Respiratory co-morbidities may enhance EDS [62] and/or upper airway instability leading to sleep disordered breathing [63] and concomitant EDS [64]. It has been demonstrated that environmental exposures are associated with increased sleep disordered breathing, possibly because of enhanced upper airway inflammation [65]. Our results show that homes requiring repairs are associated with high ESS scores. It is possible that endotoxin or mold exposure associated with dampness in these homes could result in upper airway inflammation [66, 67] thus acting by a similar mechanism. Cigarette smoking may enhance OSA [68] with consequent increased EDS. But like our finding for BMI, smoking was not a significant contributor to EDS, likely because smoking was highly prevalent in the population (78.3% current smokers; 12.2% ex-smokers). The high prevalence of non-traditional use of tobacco (smoking) is being address by a community-chosen intervention called the “Green Light Program”. The Green Light Program is an evidence-informed, community-level intervention which identifies and celebrates homes that are smoke-free. Currently, in excess of 50% of the homes in both communities are smoke-free.

Certain strengths and limitations apply to our study. Strengths include the size of the populations studied, and the consistently strong of associations with respiratory co-morbidities, indices of social determinants of health, and snoring. This study adds to a small but growing body of knowledge on sleep issues affecting Indigenous populations. A major limitation is the recall-bias of medical history due to the cross-sectional nature of the study. One of other limitations is information bias due to conducting the study in First Nation communities. That is, we hired local people to conduct the interviews and closeness among community members could lead to information bias. Response rates for household surveys and individual surveys in Community A were lower compared to Community B. Compared to Community B, Community A is spread over a much wider area of land resulting in lower response rates due to difficulties in access to the health clinic [37]. Other potential limitation of the study is the lack of validation of the ESS in First Nations people. While all the younger people in this study spoke English, on site interpretation was required for some of the older participants. It is possible that the ESS may be a culturally sensitive tool [69], with potential observer bias when used in non-Caucasian cultures.

## Conclusions

This study demonstrates relationships between respiratory co-morbidities and environmental exposures, and EDS in two First Nation communities. It is imperative to validate the use of the ESS in and with First Nations people. An important priority is to work with the communities involved to elucidate the burden of sleep disorders among First Nations people living on reserves so that the tools can be shared and used in and with other First Nation communities in Canada.

## Abbreviations

BMI: Body Mass Index
COPD: Chronic Obstructive Pulmonary Disease
EDS: Excessive Daytime Sleepiness
ESS: Epworth Sleepiness Scale
SOB: Shortness of Breath
